# Interrogation of bimetallic particle oxidation in three dimensions at the nanoscale

**DOI:** 10.1038/ncomms13335

**Published:** 2016-12-08

**Authors:** Lili Han, Qingping Meng, Deli Wang, Yimei Zhu, Jie Wang, Xiwen Du, Eric A. Stach, Huolin L. Xin

**Affiliations:** 1 Center for Functional Nanomaterials, Brookhaven National Laboratory, Upton, NY 11973, USA; 2 Institute of New-Energy Materials, Key Laboratory of Advanced Ceramics and Machining Technology, Ministry of Education (Tianjin University), School of Materials Science and Engineering, Tianjin University, Tianjin 300072, China; 3 Condensed Matter Physics and Materials Science Department, Brookhaven National Laboratory, Upton, New York 11973, USA; 4 Key Laboratory of Material Chemistry for Energy Conversion and Storage (Huazhong University of Science and Technology), Ministry of Education, Hubei Key Laboratory of Material Chemistry and Service Failure, School of Chemistry and Chemical Engineering, Huazhong University of Science and Technology, Wuhan 430074, China

## Abstract

An understanding of bimetallic alloy oxidation is key to the design of hollow-structured binary oxides and the optimization of their catalytic performance. However, one roadblock encountered in studying these binary oxide systems is the difficulty in describing the heterogeneities that occur in both structure and chemistry as a function of reaction coordinate. This is due to the complexity of the three-dimensional mosaic patterns that occur in these heterogeneous binary systems. By combining real-time imaging and chemical-sensitive electron tomography, we show that it is possible to characterize these systems with simultaneous nanoscale and chemical detail. We find that there is oxidation-induced chemical segregation occurring on both external and internal surfaces. Additionally, there is another layer of complexity that occurs during the oxidation, namely that the morphology of the initial oxide surface can change the oxidation modality. This work characterizes the pathways that can control the morphology in binary oxide materials.

## Abstract (web summary)

Understanding bimetallic alloy oxidation is key to design of hollow-structured binary oxides and their optimization for applications, e.g., as catalysts. Here the authors combine real-time imaging and chemically-sensitive electron tomography to uncover unexpected complexity in possible morphological outcomes of bimetallic oxidation.

There is increasing interest in using bimetallic particles as precursors for synthesizing hollow-structured oxides. However, this requires a comprehensive understanding of the oxidation mechanisms that occur in these materials, at the nanoscale^1^,^2^. Moreover, understanding binary oxide formation is of broad technological relevance, as these materials are finding increasing use in applications such as electrocatalysis^3^,^4^,^5^,^6^,^7^,^8^,^9^,^10^, lithium batteries^11^,^12^ and supercapacitors^13^,^14^. Additionally, these processes are also of fundamental importance to the understanding of metal alloy corrosion^15^,^16^. Despite the importance of these questions, the difficulty in resolving the spatial distribution of the structural and chemical changes that occur during bimetallic oxidation has limited progress in the field.

To solve this problem, we use mass-contrast and chemical sensitive electron tomography to visualize—in three dimensions—the structural and chemical changes that occur in Ni_2_Co nanoparticles during oxidation. This technique allows us to unravel spatially dependent chemical segregation and unscramble the nanoscale information that is lost in projected images. In conjunction with three-dimensional (3D) imaging, we use state-of-the-art environmental transmission electron microscopy (ETEM) to track the oxidation of particles in real time. Combining these techniques, we find that during oxidation a thin Ni–Co alloy oxide shell initially forms on the surface of the particle. Subsequently, metallic ions diffuse outward and are oxidized when they reach oxygen. A thin Co-rich oxide segregation layer is also formed and preserved on the outer wall of the shell. According to the Ellingham diagrams of cobalt oxides and nickel oxides, the cobalt oxide segregation phenomenon is likely driven by the difference in the oxidation potential between Co and Ni. In particular, we find that the oxidation of the metallic core slows down and eventually stagnates as the shell grew thicker. However, in 97% of the particles, after the oxide growth has stagnated, the remaining metallic core oxidizes within the already formed shell, as opposed to the system maintaining outward diffusion. Electron tomography measurements also reveal pinholes in the oxide shell. In the fully oxidized particles, chemical sensitive tomography shows that there are a large number of internal voids with Co-rich surfaces. All of these observations suggest that the transition from an outward growth mode to an internal oxidation mode is likely the result of oxygen infiltration through the pinholes. By combining real-time imaging and electron tomography, our study reveals oxidation-induced chemical segregation on external as well as internal surfaces. It also unmasks an additional layer of complexity in metal oxidation, that is, the morphology of the initial oxide surface can change the oxidation modality. This work elucidates the pathways that can control and alter morphology of hollow-structured metal oxides.

## Results

### Characterization of Ni-Co particles before and after oxidation

**Figure 1:**
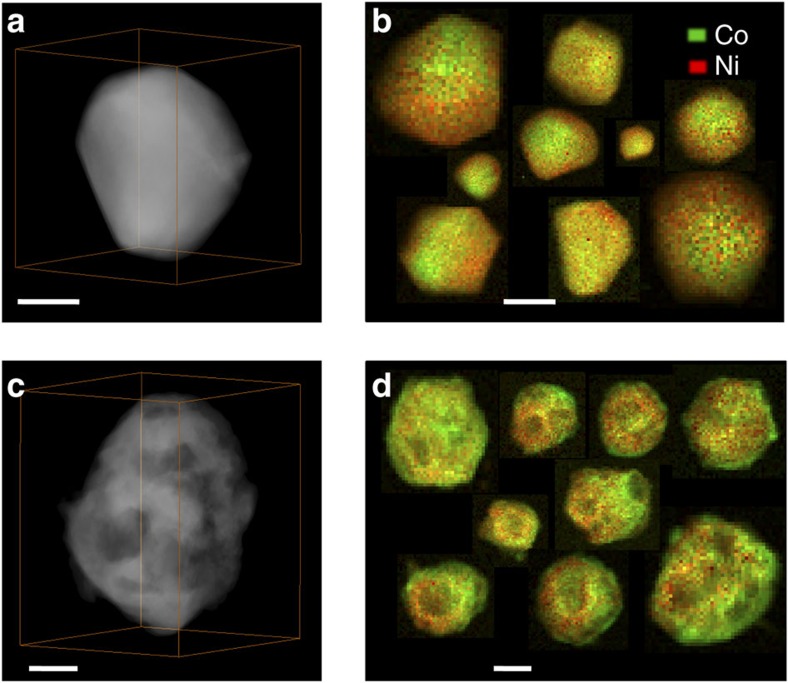
Ni–Co particles before and after complete oxidation in air. (**a,c**) HAADF-STEM tomographic reconstructions of the particles before and after oxidation, respectively. (**b**,**d**) EELS maps of the spatial distribution of cobalt and nickel in different nanoparticles (**b**) before and (**d**) after oxidation. Plural inelastic scattering was removed from the EELS spectra to exclude the influence of thickness differences. Scale bar, 50 nm.

Ni–Co particles grown on carbon nanotubes (CNTs) were synthesized by an impregnation reduction reaction strategy. The comparison of the Ni–Co particles before and after oxidation in air is shown in Fig. 1. The 3D internal structure of the particles is reconstructed by high-angle annular dark-field scanning transmission electron microscopy (HAADF-STEM) tomography. Figure 1a presents the volume rendering of the 3D reconstruction of a pristine particle. It shows that the pristine particle has a solid structure with facet exposure. (The two-dimensional morphology and particle distribution are also shown in Supplementary Fig. 1a,b). The STEM-electron energy loss spectroscopic (EELS) mapping in Fig. 1b reveals intermixing of nickel and cobalt in an ensemble of particles, suggesting Ni–Co alloy formation. The atomic ratios between Ni and Co are calculated by using the continuum portion of the L_2,3_ edges of nickel and cobalt, with removal of plural scattering from each particle shown in Fig. 1b. The statistics are shown in Supplementary Fig. 1d and the average measured ratio is Ni:Co=1.90±0.09, close to the nominal value, 2. The indexed peaks of the X-ray diffraction pattern in Supplementary Fig. 1e correspond to the face-centered cubic (fcc) structure, which is consistent with the analysis of the selected area electron diffraction (SAED) pattern in Supplementary Fig. 1c. This indicates that Co atoms take random substitutional positions, resulting in the formation of a fcc Ni_2_Co alloy^17^. After heating at 450 °C in air for an hour, the Ni_2_Co particles were fully oxidized. Figure 1c shows that the 3D nanostructure of the particles features a series of internal cavities and voids. The compositional distribution of nickel and cobalt for a large number of nanoparticles is shown in Fig. 1d. It is worth noting that the oxide particles are encased by a Co-rich surface layer, indicating the presence of cobalt segregation.

### *In situ* environmental study of Ni–Co oxidation

**Figure 2:**
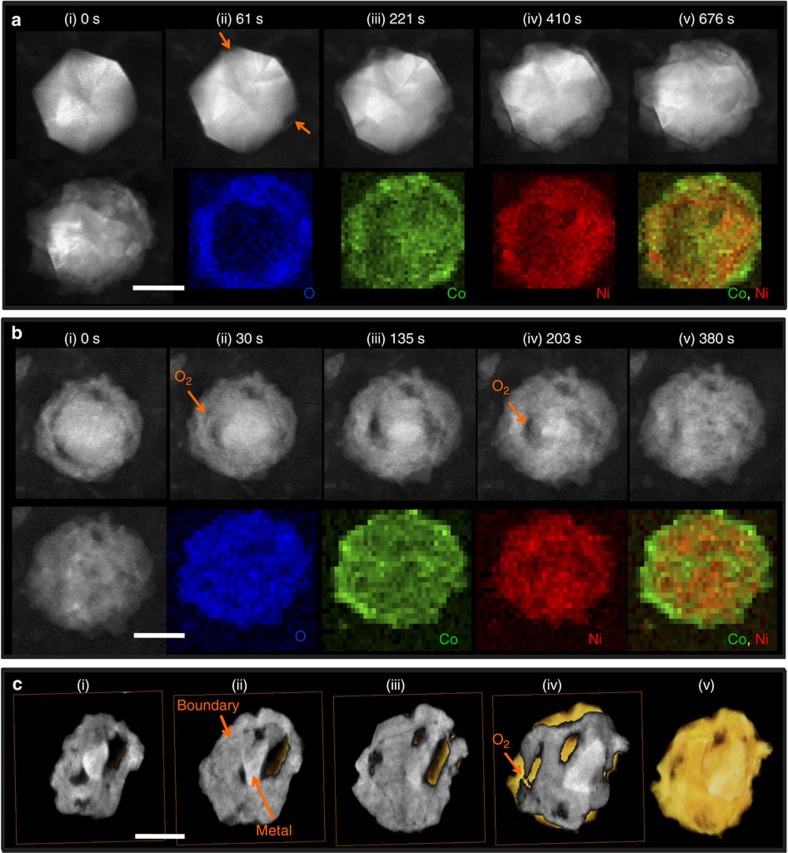
*In situ* observation of structural and compositional changes during Ni_2_Co oxidation. (**a**) *In situ* ADF-STEM images of one particle in the first oxidation stage show the elemental migration from the inside of the particle to the surface, resulting in the formation of a core-shell structure. The *in situ* EELS mapping of O, Co and Ni elements shows that the shell consists of a Ni and Co binary oxide with a few Co oxide nanodomains on the outside. (**b**) *In situ* ADF-STEM images of the particle during further oxidation show the oxidation of the inside core. The O, Co and Ni elemental distribution in the fully oxidized sample shows that the particle is encased by Co-rich oxides. (**a**) and (**b**) are from different particles. (**c**) A series of cross-sections and isosurfaces created by the electron tomography technique allow visualization of the internal structure of partially oxidized particle. Scale bar, 50 nm.

Figure 2 presents the results from an environmental TEM study of the structural and compositional evolution of Ni_2_Co particles during oxidation at elevated temperatures. Real-time observations show that the oxidation progresses in two stages. The heating profiles in the two stages are shown in Supplementary Fig. 2. In the first stage, the particle was partially oxidized to form a core-shell structure, as illustrated by Supplementary Movie 1. Figure 2a presents a few still images of a single particle at selected times showing its structural evolution during oxidation. After oxidation for 61 s at 400 °C, oxidation started at the two vertices of the particle, resulting in the formation of two cavities (indicated by their lower intensity in the ADF-STEM images, see the arrows in Fig. 2a(ii)). This preferential nucleation from edges and vertices was also observed in other particles. With continued oxidation, additional cavities started to appear and an outer oxide layer formed simultaneously. This indicates atoms were removed from the metallic core and diffused through the oxide layer to combine with oxygen, as described by the Kirkendall effect^18^,^19^. However, the oxidation of the metallic core slowed down and stagnated as the shell grew thicker. After the first stage of oxidation, *in situ* STEM-EELS mapping of the particle was performed. Figure 2a shows that a thin Co-rich oxide layer segregates to the outer wall of the shell, leaving a Ni-rich oxide layer on the inner wall. This is in significant contrast to the elemental distributions on the surfaces of the pristine particles shown in Supplementary Fig. 3.

The oxidation was continued for a second stage by raising the temperature further, in steps. The Z-contrast STEM time sequence images are shown in Fig. 2b and Supplementary Movie 2. After heating at 500 °C for 18 s and at 520 °C for 2 s in oxygen, the metal core started to be oxidized inside the previously formed oxide layer and another shell-like feature emerged internally, as indicated by the orange arrows in Fig. 2b(ii). As the oxidation progressed, the shell-like feature extended further and formed a double shell in the particle, as shown in Fig. 2b(v). The EELS maps after complete oxidation in Fig. 2c presents the O, Co and Ni elemental distribution: from this we see that the particle is wrapped by a Co-rich oxide layer. The crystal structure is determined from the SAED pattern in Supplementary Fig. 4b, which can be indexed to be consistent with the Ni_x_Co_3−x_O_4_ spinel structure.

This oxidation of the metallic core on the inside of the shell contradicts the prevailing wisdom regarding how the Kirkendall effects works in metallic oxidization systems, that is, that a metallic particle would hollow out during oxidation^18^,^20^. However, we repeatedly observed this internal oxidation phenomenon (another *in situ* observation with similar effects is presented in Supplementary Fig. 5). It has been reported that Pb nanoparticles do not form internal nanovoids during oxidation because in this system, oxygen anions diffuse faster than the lead cations^21^. In the Ni–Co systems, however, their mono-elemental nanoparticles both develop hollow structures during oxidation^18^,^22^. Therefore, it is less likely that the observed internal oxidation phenomenon is a result of fast inward oxygen transport via diffusion.

To rule out the possibility of inward diffusion of oxygen through the initial oxide shell quantitatively, we compared the temperature-dependent self-diffusivity of the cations and the oxygen anion in NiO and CoO using data extracted from the literature^23^,^24^,^25^,^26^,^27^,^28^ (see Supplementary Fig. 6). We found that in nickel and cobalt oxides, the diffusion coefficient of oxygen is several orders of magnitude lower than the diffusion coefficient of their corresponding cations in bulk as well as along grain boundaries. It is very unlikely that the oxygen could reach the metals in the core by diffusing through the oxide shell under our temperature conditions. Therefore, we suspect that this departure from the regular Kirkendall hollow structure could likely be attributed to the formation of pinholes in the oxide shell, that is, oxygen molecules could infiltrate the interior of first shell and directly oxidize the metals in the core^29^. However, real-time imaging only provides projection images. Nanoscale pinholes overlap with other materials along the projection direction making it difficult to pinpoint their locations. To reliably visualize the 3D structure of the oxidized particles without ambiguity, we reconstructed a partially oxidized particle with electron tomography using ADF-STEM signals in the ETEM, immediately after we quenched the reaction by lowering the reaction temperature to room temperature.

The 3D reconstruction of the particle is presented in Fig. 2c and Supplementary Movie 3. Because we used ADF-STEM signals for tomographic reconstruction, the intensities of the reconstructed tomograms are directly interpretable, with higher intensities reflecting higher atomic mass density. As shown in the progressive cross-sectional views of the particle, there are two distinct levels of intensities. The lower intensity level is associated with the oxide and the higher intensity level represents the unreacted metal. (This is because the metal has higher packing density of nickel/cobalt atoms than the oxide.) Looking closely at the reconstructed cross sections in Fig. 2c, there exists an obvious low-intensity boundary between the inner oxides and the outer oxide shell. This indicates that the oxidation of the particle had progressed past stage one. From the reconstruction, we also found that the outer oxide shell is not continuous but has holes in it, as indicated by the arrow in Fig. 2c(iv).

### *Ex situ* validation

**Figure 3:**
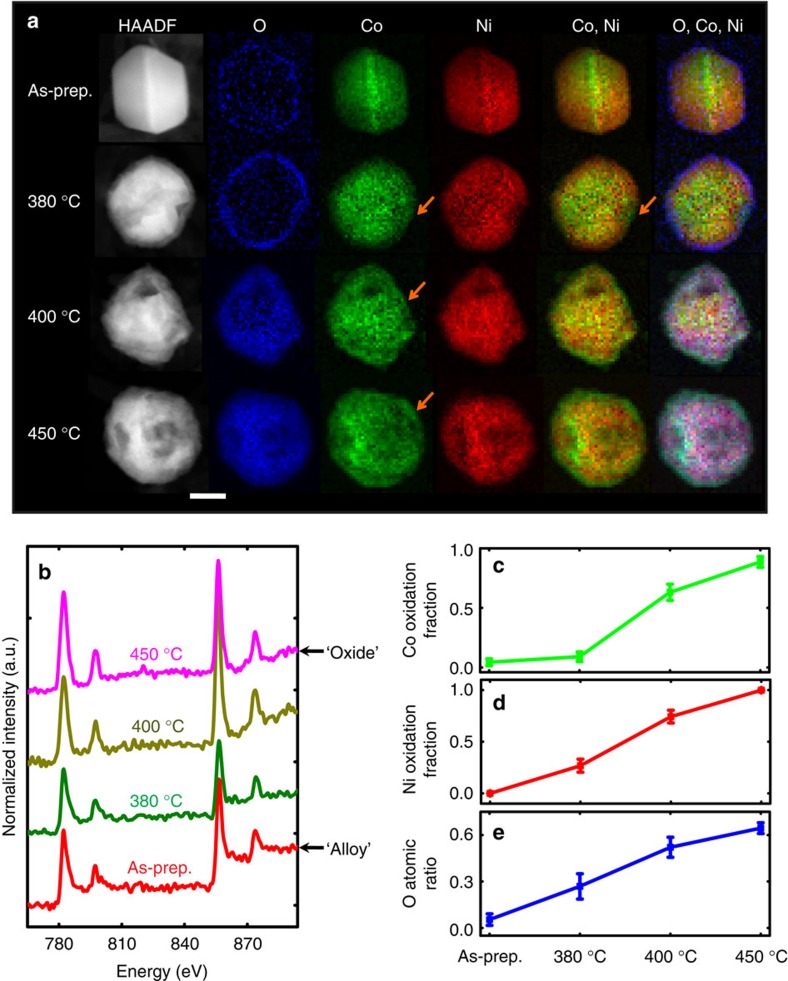
*Ex situ* observation of Ni_2_Co particle as a function of oxidation temperature. Data are from particles oxidized under air for 1 h at various temperatures. (**a**) HAADF-STEM images and EELS mappings. The EELS mapping is shown after removing plural scattering. Scale bar, 50 nm. (Note these are four different particles from the samples at different reaction temperatures.) (**b**) Four EELS spectra of Co and Ni L_2,3_ edges, respectively, extracted from the EELS images of the four particles in (**a**). (**c**) Oxidized Co fraction in oxidized and metallic Co calculated from (**b**). (**d**) Oxidized Ni fraction in oxidized and metallic Ni calculated from (**b**). (**e**) Oxygen atomic ratio in O, Co and Ni elements calculated from (**b**).

It is worth noting that electron beams can cause knock-on damage, local heating and induced coalescence. To exclude the influence of the electron beam, we performed *ex situ* quantification of the temperature-dependent changes in structure, composition and valence state of Ni_2_Co particles during oxidation in air. As shown in Fig. 3a, the structural and compositional evolution as a function of temperature was recorded by HAADF-STEM images and STEM-EELS mappings.

For samples heated at 380 °C for an hour, the bimetallic oxidation was initiated on the surface of the particle, and a thin Ni–Co oxide layer of ∼5–10 nm thickness was formed. The composition of nickel and cobalt in the oxide shell have the bulk ratio of 2:1, except around a cavity at a vertex location indicated by the arrow. The shell around the metal leaching site—the cavity formation location, indicated by the arrows—has a higher composition of cobalt than the rest of the oxide layer. This suggests that once the Kirkendall effect is activated, it may be easier for more Co atoms than Ni atoms to migrate through the oxide shell to form Co-rich oxides.

For samples heated at 400 °C for an hour, more metal atoms were removed from the metal core and permeated through the oxide layer to combine with oxygen, and thus the cavity increased in size and the oxide shell became thicker. In addition, rather than having a few Co-rich domains, the entire shell was coated with a thin Co-rich oxide layer. The nickel and cobalt concentration lines profiles in Supplementary Fig. 7e show this segregation quantitatively. The EELS spectra in the Co-dominated and Ni-dominated volumes in Supplementary Fig. 7f indicates the co-existence of cobalt and nickel in the two volumes, suggesting cobalt and nickel atoms can inter-diffuse to form Ni–Co oxide.

For samples heated at 450 °C for an hour, the particle was completely oxidized. Cobalt oxide segregation can also be clearly observed. The SAED pattern of this material is in agreement with the pattern expected for a Ni_x_Co_3−x_O_4_ spinel structure (Supplementary Fig. 8). This is consistent with the crystal structure formed in the ETEM.

To correlate the structural evolution with the fraction of metallic oxidation, we used EELS to monitor the electronic structure changes of cobalt and nickel. Figure 3b shows the near-edge fine structures of the Co and Ni L_2,3_ edges recorded from four samples that were treated at room temperature, 380, 400 and 450 °C. Since the as-prepared material is a nickel and cobalt alloy (based on the analysis of X-ray diffraction and SAED patterns in Supplementary Fig. 1), the as-prepared spectrum can be used as metallic Co ‘0' and Ni ‘0' reference spectra (‘fingerprints'). Similarly, the sample after full-oxidation at 450 °C can be approximately assigned to be the fully oxidized-Co and fully oxidized-Ni fingerprints. The L_2,3_ near-edge fine structures of metals with an average valence state between these two end points can be decomposed into a linear combination of the two fingerprints. To improve the purity of the fingerprints, they were in turn refined using a Multivariate Curve Resolution method^30^. The corresponding decomposition coefficient of the oxidized Co component reflects the fraction of oxidation (Fig. 3c,d). We see that the oxidized fraction increases with temperature. This is further confirmed by the change in oxygen fraction as a function of reaction temperature in Fig. 3e.

**Figure 4:**
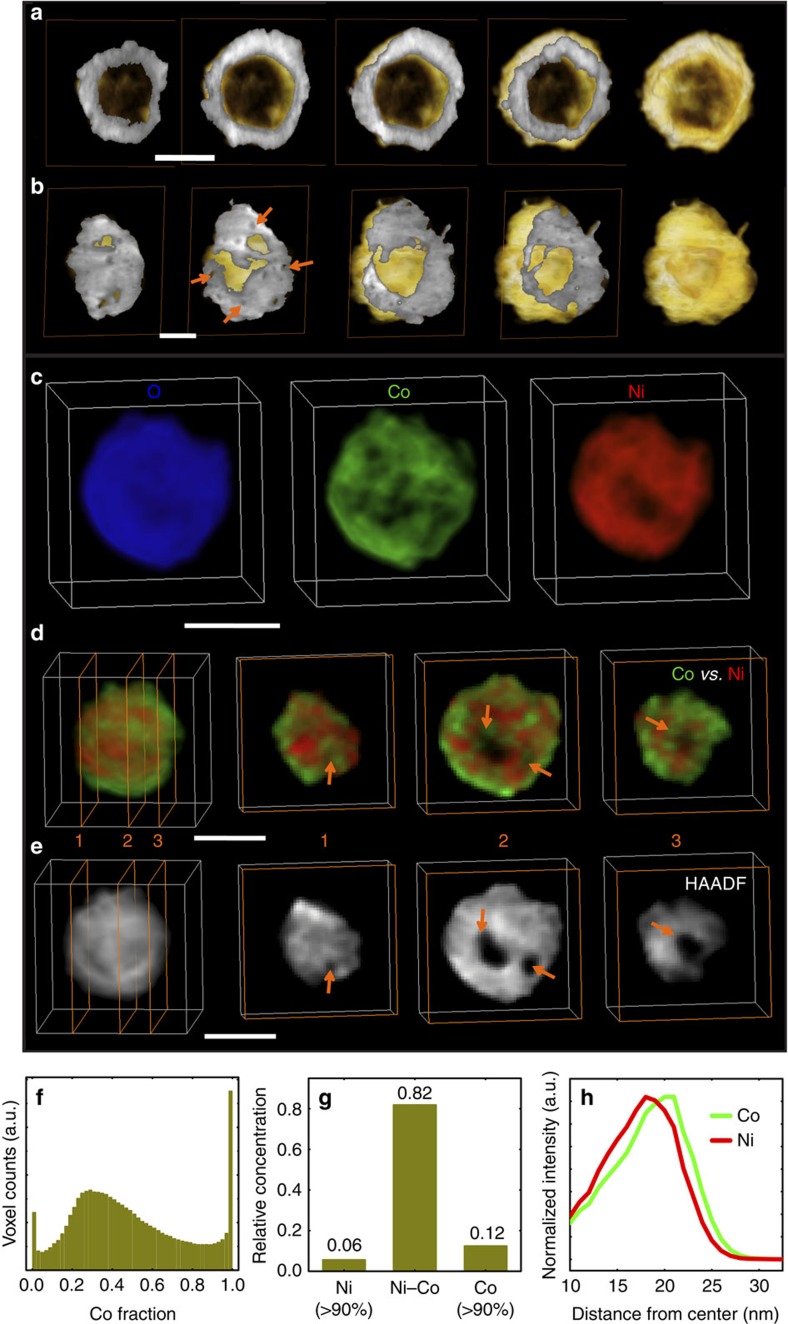
3D structure, elemental mapping and elemental-association data for completely oxidized particles. (**a**) 3D structure of a Type I particle, with a solid shell and large hollow volume fraction (44.79%). (**b**) 3D structure of a Type II particle, which has a nanoporous shell and small hollow volume fraction (11.52%) when compared with (**a**). (**c**) 3D elemental distribution after removing plural scattering. (**d**,**e**) Sequential cross sections and 3D rendering of the Ni and Co color-mixed map and HAADF-STEM map, respectively. Comparing (**d**) with (**e**) illustrates that a large number of Co element nanodomains are spatially separated from Ni and are concentrated on the outside of the shell and around the holes. Scale bar, 50 nm. (**f**) The fractional distribution of Co voxel counts in Co and Ni. (**g**) The relative concentrations of 3D elemental associations. (**h**) Elemental distribution as a function of the distance from nanosphere center calculated using 3D STEM-EELS data.

Two different morphologies of particles are observed in the sample after complete oxidation at 450 °C in air for an hour. Figure 4a,b shows the 3D structure of these two types of particles. The 2D projection views are shown in Supplementary Fig. 9; however, those images cannot directly visualize the internal structure of the particles. The consecutive cross-sectional images and 3D rendering in Fig. 4a show that the first type of particle has a solid shell with one single void inside. This type of particle (Type I) are smaller in size (<150 nm when oxidized) and has a 3% occurrence in the product. This type of particles has a fully hollowed structure, which results from the regular Kirkendall effect. The presence of the solid oxide shell prevents oxygen infiltration, so that metal elements have to diffuse through the oxide layer to be oxidized on the surface. This leads to the formation of a large void inside the particle. However, the second type of particle (Type II) dominates the product. It has a porous shell with a lower void volume fraction when compared with the first type, as shown in Fig. 4b. The formation of pinholes in the oxide shell could be attributed to the islanded growth effect driven by the non-wettability, as well as the strain relaxation due to the lattice mismatch between the metal core and the metal oxide shell during the oxidation process^2^,^31^. The formed pinholes can facilitate the infiltration of oxygen molecules at reaction conditions; consequently, the metal can be oxidized inside the shell, which however was not observed in the oxidation of their parental mono-metallic structures, such as crystalline nickel nanospheres and cobalt nanospheres, because conformal shells were formed during their oxidation^22^,^32^. These results are consistent with the speculation that oxygen can permeate through the shell, as was discussed in during the explanation of the *in situ* TEM experiment shown in Fig. 2b,c.

Figure 4c–h presents 3D elemental distributions of a fully oxidized particle formed by oxidation at 450 °C in air for an hour, reconstructed using chemical sensitive electron tomography (STEM-EELS tomography). The color maps of the individual elements in the 3D coordinates are directly visualized in Fig. 4c as well as in Supplementary Movie 4. The internal elemental distributions are visualized by slicing through element-specific reconstructions (see Fig. 4d and Supplementary Movie 5). The Z-contrast tomography reconstruction in Fig. 4e demonstrates that there are cavities and voids in the oxide particle that cannot be directly observed in projection images. By comparing Fig. 4d,e, it is easy to identify that there is a higher concentration of cobalt on the outer surface of the shell as well as around the internal surfaces of the voids/holes. This is in significant contrast to the Type I particles, in which the inner surface has less cobalt (Supplementary Fig. 7d). This again strongly suggests that oxygen has infiltrated the particle and directly oxidized the remaining metallic core, resulting in Co-rich surfaces on the internal voids.

To quantify whether or not Co and Ni are segregated or mixed, we calculated the volume fraction of the particle as a function of cobalt composition (shown in Fig. 4f). The resulting composition-volume histogram has a continuous distribution: this indicates that the segregation of cobalt has a gradated profile (in agreement with Supplementary Fig. 7e). This is also indicated by the statistical analysis of Fig. 4g, which shows that the Ni–Co ‘association' is high (at 82%). By association, we mean that a given voxel of element A contains both Ni and Co at a concentration ratio between 1:9 and 9:1. This again shows that the composition of the particle is dominated by Ni–Co bimetallic spinel oxides. The radially averaged elemental distribution (calculated using EELS tomography data) shows that there is an outward segregation of cobalt (Fig. 4h), which is consistent with the observations in Figs 1d and 2b.

## Discussion

To understand the Co and Ni elemental segregation and association seen in our particles during oxidation, we performed three different types of analysis, the Ellingham diagram (a plot of Gibbs free energy versus temperature), Mott and Cabrera's theory and the phase diagram of CoO_x_–NiO_x_ system at one atmosphere pressure.

**Figure 5:**
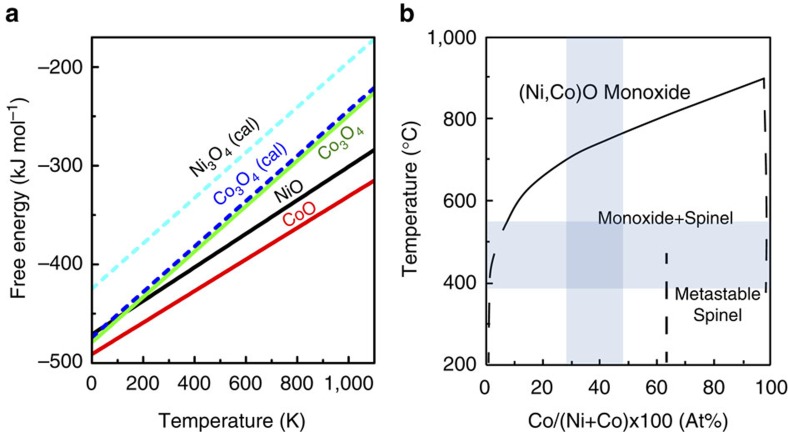
Ellingham and phase diagrams of the cobalt oxide and nickel oxide system. (**a**) Ellingham diagrams (a plot of Gibbs free energy versus temperature) of cobalt oxides and nickel oxides at one atmosphere pressure. The curves with dashed lines are Ellingham diagrams calculated using generalized gradient approximations (GGA). (**b**) The phase diagram of CoO_x_–NiO_x_ system at one atmosphere pressure adapted from ref. 40. The areas shadowed in blue correspond to the reaction temperature and the Co atomic ratio in pristine particles relevant to our experiments.

For the first analysis, the Gibbs free energy of a metal-oxidized reaction is a measure of the driving force, which drives the metal outward through diffusion to combine with oxygen^33^. The Ellingham diagrams in Fig. 5a show the experimentally determined free energy of CoO is more negative than NiO. Similarly, the free energy of Co_3_O_4_ is more negative than the calculated energy of Ni_3_O_4_ (calculated with the generalized gradient approximation (GGA+U) implementation of density functional theory due to the lack of naturally occurring Ni_3_O_4_). We also find that the formation energy of NiCo_2_O_4_ is lower than that of Ni_2_CoO_4_. These indicate that the driving force of Co oxidation is larger than Ni.

To understand the oxidation kinetics, in the second analysis, we introduce Mott and Cabrera's theory for thin oxides layer^33^,^34^ and Wagner's theory for thick oxides layer^35^, which can quantitatively explain the rate of oxide formation on a metal surface. In the Mott and Cabrera's theory, the ionic diffusion current as a function of existing oxide layer thickness, L, is:


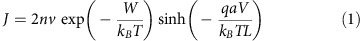


where *n* is the ionic number per unit area, which are in a position to jump the rate-limiting energy barrier *W*, *v* is the ionic attempt frequency, 2*a* is the ionic jump distance, *q* is the charge per particle of the diffusion ionic species, *k*_*B*_ is the Boltzmann constant, *T* is the absolute temperature, *L* is the thickness of oxide layer and *V* is the electrical contact potential. For our case, we can think that *n*, *v*, 2*a* and *q* are same for Co and Ni. For the thin oxides layer, the electrical contact potential *V* is a key to determine the Co or Ni diffusion rate. The *V* from Mott and Cabrera's theory^33^,^34^ is


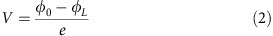


where *e* is the magnitude of the electronic charge, *ϕ*_0_ is the metal-vacuum work function, *ϕ*_*L*_ is the adsorption energy of an oxygen ion O^−^ on the surface of oxide. Mott and Cabrera's theory tell us that *V* is negative in sign. The negative voltage leads to electron transfer from metal to oxygen. The adsorption energy *ϕ*_*L*_ is a constant for an oxide of fixed composition and the work functions of Co and Ni are 5.0 and 5.15 eV, respectively^36^. Therefore, *V* is more negative for Co than that for Ni. It means Co ions are more facile to migrate through the oxide shell to combine with oxygen than Ni ions if other variables are assumed identical.

The rate-limiting energy barrier *W* determines the rate of diffusion of metal ions. Wagner's theory of thick oxide film growth provides a means by which the rate of oxide film growth relates to the diffusion coefficients^35^. Using the temperature dependence of the diffusion coefficients documented in the literature^26^,^37^,^38^,^39^, we found that the diffusion coefficients of Co and Ni in Ni–Co (with 30.3 at.% Co) alloy are 8.11 × 10^−20^ and 1.51 × 10^−21^ cm^2^ sec^−1^, respectively, at 450 °C, and those of Co in CoO and Ni in NiO are 1.22 × 10^−14^ and 1.94 × 10^−20^ cm^2^ sec^−1^, respectively, at the same temperature. These diffusion coefficients indicate that it is easier for cobalt to migrate to the surface than nickel does. It in turn explains why the Co-rich segregation layer forms on the surfaces of the particles.

In the third analysis, the high Ni–Co association in oxides can be understood by considering the binary phase diagram of the CoO_x_–NiO_x_ system at one atmosphere pressure, as shown in Fig. 5b (ref. 40). From the diagram, the cobalt oxide has a high solubility in nickel oxide at reaction temperatures (from 380 to 550 °C). This well explains why the product after full oxidization in our system has high Ni–Co association.

In conclusion, to understand the spatially dependent reaction pathways of bimetallic oxidation, we have used environmental TEM, mass-contrast and chemical sensitive tomography to visualize the oxidation of Ni_2_Co particles. Both *in situ* chemical mapping and *ex situ* chemical tomography demonstrate a graded segregation of cobalt and nickel oxides, with more cobalt sitting close to the surfaces that are exposed to oxygen. Apart from the segregation, we observed an unexpected deviation from the traditional Kirkendall-induced hollowing process. The metal core inside the oxide shell can be oxidized from within, rather than maintaining an outward diffusion pathway. Following this new pathway, we found that both the external oxide shell and the internal oxides are porous in nature. Our chemical sensitive tomography resolved that all internal surfaces on the inner voids are rich in cobalt, if the particle undergoes this special pathway. This is in significant contrast to the chemical profile expected from the regular Kirkendall-induced hollow structure—only the external surfaces are rich in cobalt. These findings strongly suggest possibility of infiltration of oxygen into the hollow structure at the latter stages of oxidation, due to pinholes in the initially formed oxide shell.

## Methods

### Ni_2_Co/CNTs preparation

An impregnation reduction reaction strategy was developed to synthesize bimetallic Ni_2_Co alloy particles supported on multi-walled CNTs. In detail, 54 mg CoCl_2_·6H_2_O, 108 mg of NiCl_2_·6H_2_O and 60 mg of multi-walled CNTs were added into a 25 ml beaker. Then, 10 ml of purified water was poured into the beaker. A thick slurry was formed by exchanging magnetic stirring and ultrasonic dispersion at 60 °C to evaporate water. Subsequently, the composite was transferred into a vacuum drying oven at 40 °C overnight to guarantee a completely drying. After milling in agate mortar, the resulting powder was annealed in 350 °C for 3 h under H_2_ atmosphere.

### Ni_2_Co/CNTs oxidized in air

The Ni_2_Co/CNTs powder synthesized by the procedures above was ultrasonically dispersed in methanol. The mixture was dropped on the 200 mesh TEM nickel grid with grapheme-enhanced lacey carbon. It was placed in a furnace and oxidized at the given temperature (from 380 to 450 °C) in air. After 1 h, we removed the nickel grid from the furnace and left it to cool to room temperature for TEM characterization.

### *In situ* oxidation of Ni_2_Co/CNTs in TEM

The as-prepared Ni_2_Co/CNTs was dispersed in methanol and spread on a nonporous amorphous silicon nitride TEM membrane. The *in situ* oxidation experiments were performed in a dedicated field-emission environmental TEM (FEI Environmental Titan 80–300). The ETEM is equipped with an objective lens spherical aberration corrector and controlled gas pressure around the sample using a differential pumping system. All of the oxidation experiments for *in situ* TEM were imaged at 300 kV. A pure O_2_ atmosphere was set at 0.2 torr around the sample and flowed for at least 20 min before heating. *In situ* heating was performed using a DENSsolutions MEMS device-based heating holder. The local heating on the MEMS device is based on the Joule heating of a micro-patterned metal heater. The resistance of the heater has a linear response to temperature in the range from room temperature up to 800 °C. The measurement of the relationship between the temperature and the resistance of the heater was performed prior to experiments by the manufacturer. (The temperature was measured using Raman scattering of the silicon substrate). In the ETEM experiment, the temperature was recorded using the Digiheater software by measuring the heater resistance.

### Annular dark-field STEM tomographic reconstruction

The annular dark-field scanning transmission electron microscopy (ADF-STEM) tilt series was acquired from −70° to 70° at one- or two-degree intervals. No noticeable mass loss or radiation damage was observed during the acquisition process. The acquired tilt series was first coarsely aligned using cross correlation. The final registrations were made manually using a Matlab script package (e-Tomo) written by R. Hovden (Muller group, Cornell) with contributions from H. Xin. The 3D dataset was reconstructed by the simultaneous iterative reconstruction algorithm implemented in Matlab. Ten iterations were used for the final refinement.

### STEM-EELS tomographic reconstruction

EELS images and mass-thickness HAADF-STEM images were sequentially acquired at 7° tilt intervals from −71.4° to 69.7° on a JEOL 2100F equipped with a Gatan Tradium EELS Spectrometer. In the tilt series, 30 × 30 pixel core-loss (from 400 to 1014.4 eV) EELS images were recorded with a 0.3 eV per channel dispersion and at 0.1 s per pixel, followed by the collection of zero-loss EELS images under the same conditions except for energy shift to 0 eV and the change of exposure time to 0.001 s per pixel. The measured EELS near-edge signal maps were processed by power-law subtraction in conjunction with a division by the Beer's law factor, that is, 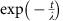 extracted from the zero-loss map, to remove the nonlinearity of the signal^41^. The 3D tomograms were reconstructed by the multiplicative simultaneous iterative reconstruction technique.

### Density functional theory calculations

The calculations were performed using the ABINIT simulation software within the projector augmented-wave approach with the Perdew–Burke–Emzerhof GGA. Hubbard U term of 3.4 and 6.0 eV is, respectively, adopted for Co and Ni in accordance with the previous testing^42^,^43^.

### Data availability

The data that support the findings of this study are available from the corresponding author upon request.

## Additional information

**How to cite this article:** Han, L. *et al*. Interrogation of bimetallic particle oxidation in three dimensions at the nanoscale. *Nat. Commun.* **7,** 13335 doi: 10.1038/ncomms13335 (2016).

**Publisher's note:** Springer Nature remains neutral with regard to jurisdictional claims in published maps and institutional affiliations.

## Supplementary Material

Supplementary Information Supplementary Figures 1-9

Supplementary Movie 1 *In situ* observation of oxidization of a Ni-Co particle in the first stage. The *in-situ* image series were acquired in the environmental TEM using the annular dark-field STEM imaging mode. The frames were aligned to correct sample drift. The movie is accelerated by 43 times.

Supplementary Movie 2 *In situ* observation of oxidization of Ni-Co particles in the second stage. The *in-situ* image series were acquired in the environmental TEM using the annular dark-field STEM imaging mode. The frames were aligned to correct sample drift. The movie is accelerated by 32 times

Supplementary Movie 3 Progressive cross-sectional view of the 3-D reconstruction of a partially-oxidized particle. The *in-situ* tomography tilt series were acquired in the environmental TEM using the annular dark-field STEM imaging mode.

Supplementary Movie 4 Rotation of the volume rendering of Co, Ni as well as Co and Ni distributions in a fully oxidized particle. The distributions were reconstructed from STEM-EELS tomography tilt series.

Supplementary Movie 5 Progressive cross-sectional view of the 3-D reconstruction of the Co and Ni distribution in a fully oxidized particle. The particle is the same one in Supplementary Movie 4.
