# Supplementary material for: Interrogation of bimetallic particle oxidation in three dimensions at the nanoscale
Source: Nat Commun. 2016 Dec 8;7:13335. doi: 10.1038/ncomms13335 (PMC5155145; doi:10.1038/ncomms13335)
Supplement: Supplementary Information — Supplementary Figures 1-9 [file ncomms13335-s1.pdf]

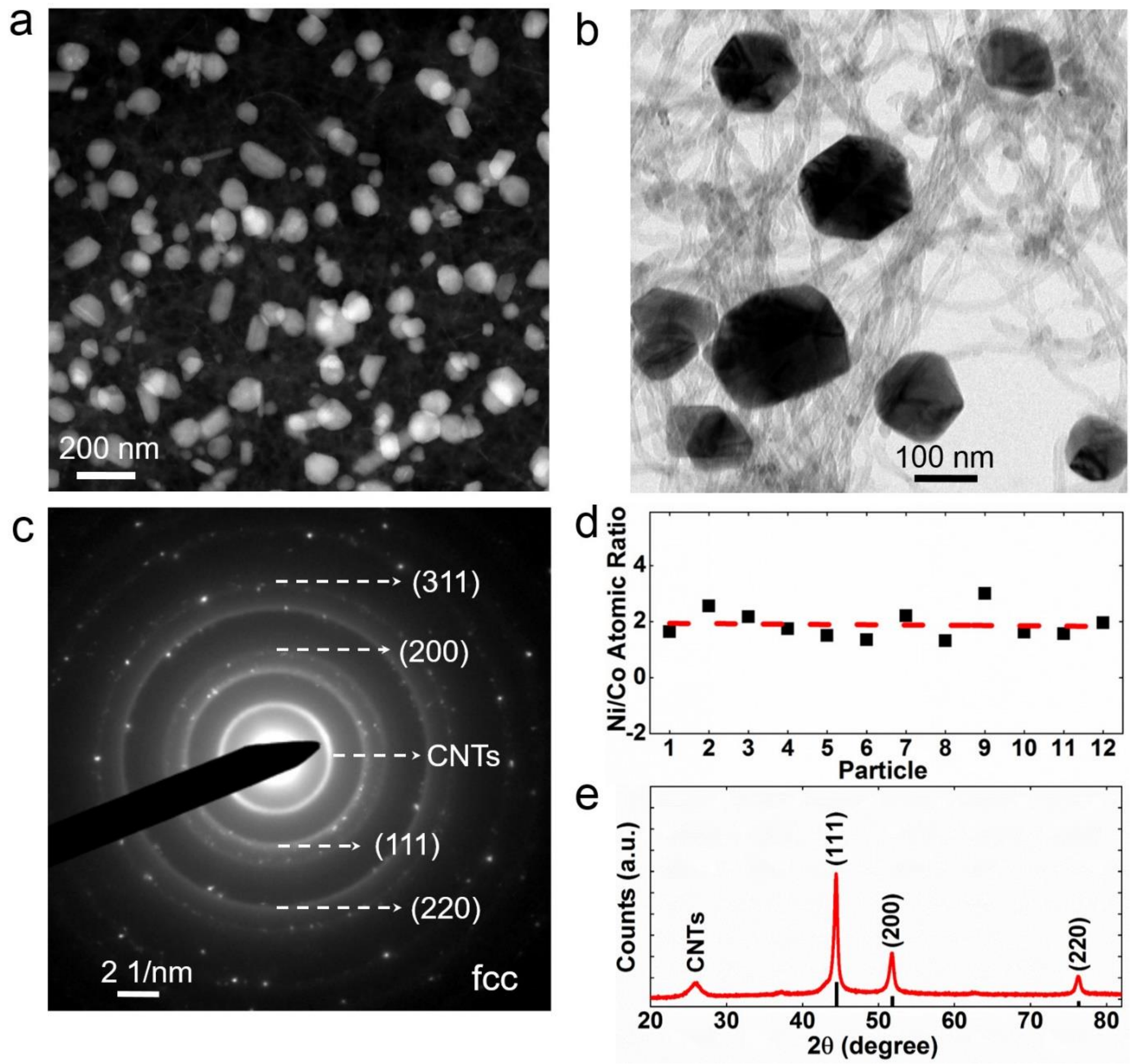

Supplementary Figure 1 | Characterization on pristine sample. (**a**) Scanning transmission electron microscopy (STEM) image; (**b**) transmission electron microscopy (TEM) image; (**c**) selected area electron diffraction (SAED) pattern; (**d**) compositional atomic ratios of Ni and Co in different particles; the average atomic ratio calculated from the fitting curve in (**d**) is Ni:Co = 1.90 ± 0.09, close to the nominal value, 2. (**e**) X-ray diffraction (XRD) pattern; the indexed peaks are consistent with those of the Ni 04-0850 PDF card, which indicates that Co elements are dissolved within the Ni to form Ni-Co alloy.

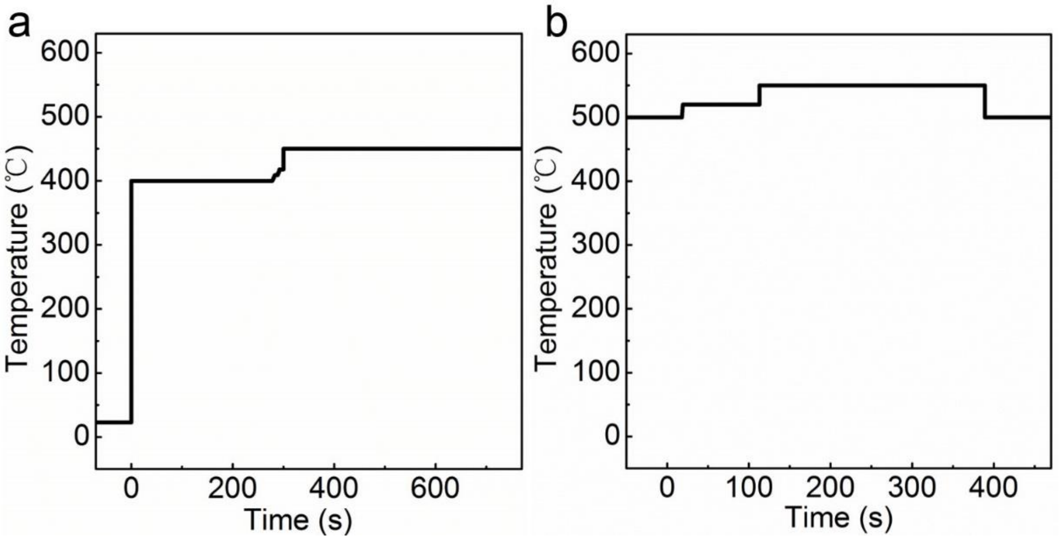

Supplementary Figure 2 | Heating profiles for the two stages of the *in situ* TEM study. (**a**) Stage I; (**b**) Stage II

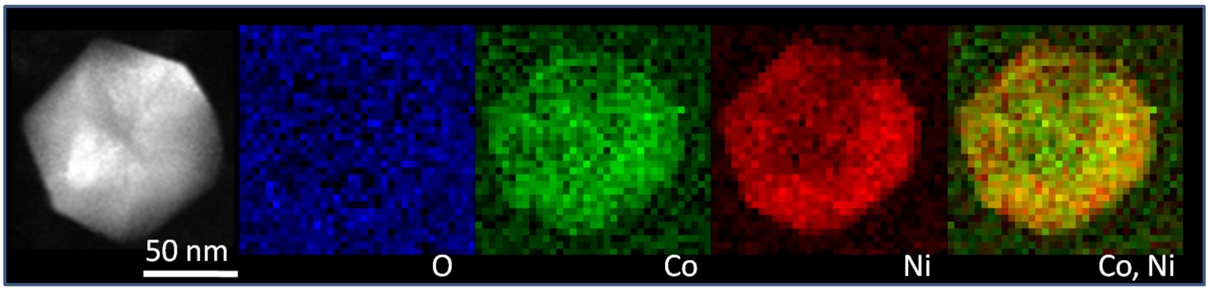

Supplementary Figure 3 | Electron energy loss spectroscopic (EELS) elemental mapping of the particle before *in situ* oxidation in the environmental TEM (ETEM). The unphysical dip in intensity in the Co and Ni maps at the center and upper part of the particle is due to diffraction contrast.

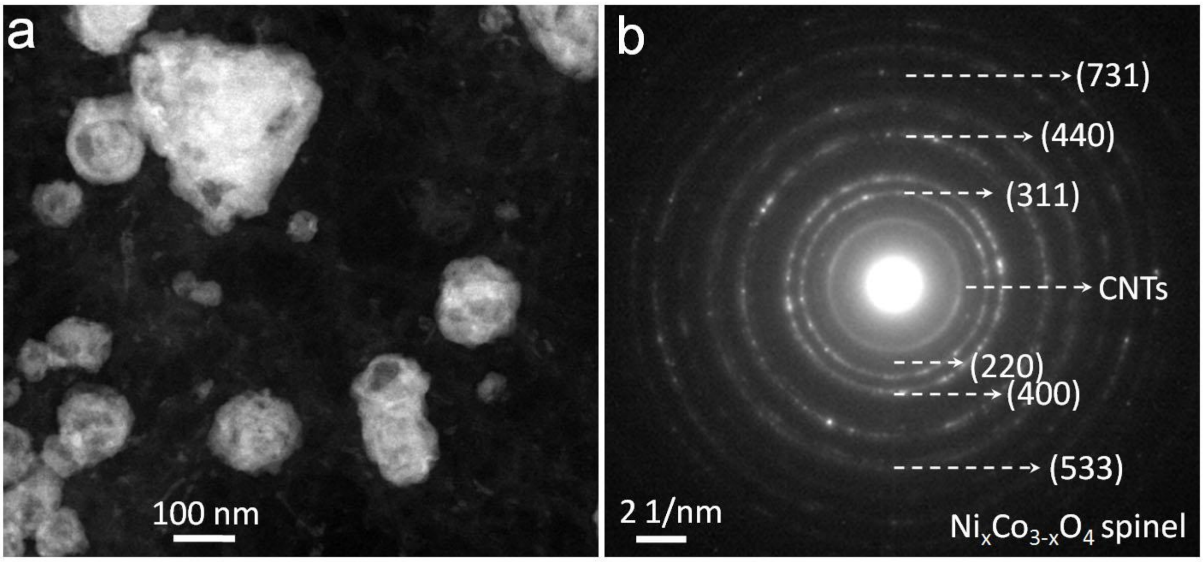

Supplementary Figure 4 | Characterization of morphology and crystal structure of the Ni_2Co sample after complete oxidation *in situ*. (**a**) STEM image, (**b**) SAED pattern. The SAED pattern indicates that the product of the oxidation process is the spinel structure.

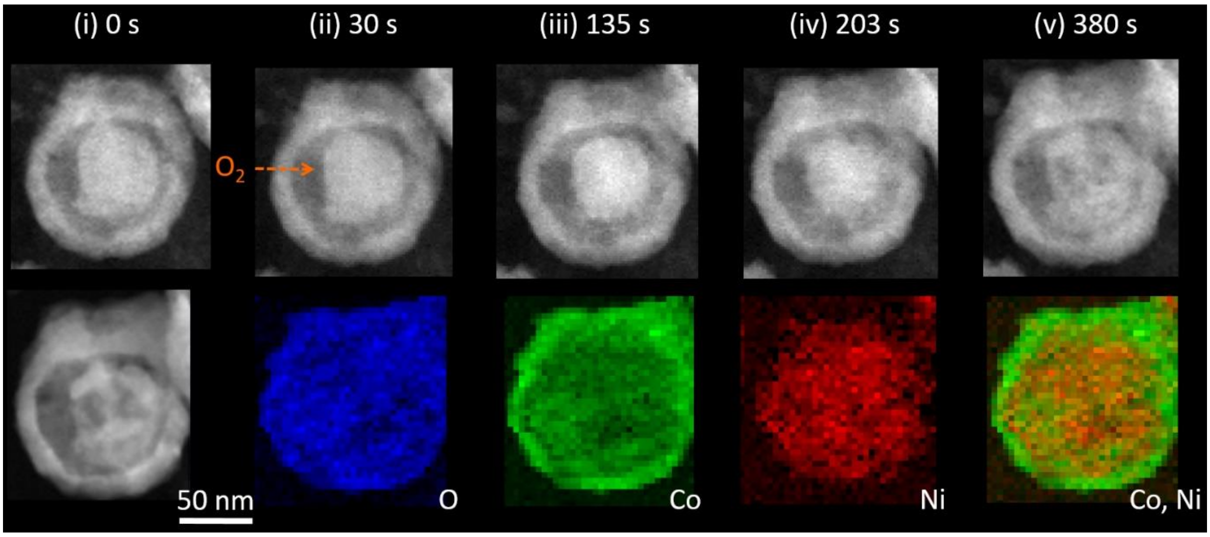

Supplementary Figure 5 | *In situ* observation of structural and compositional changes of another particle during oxidation.

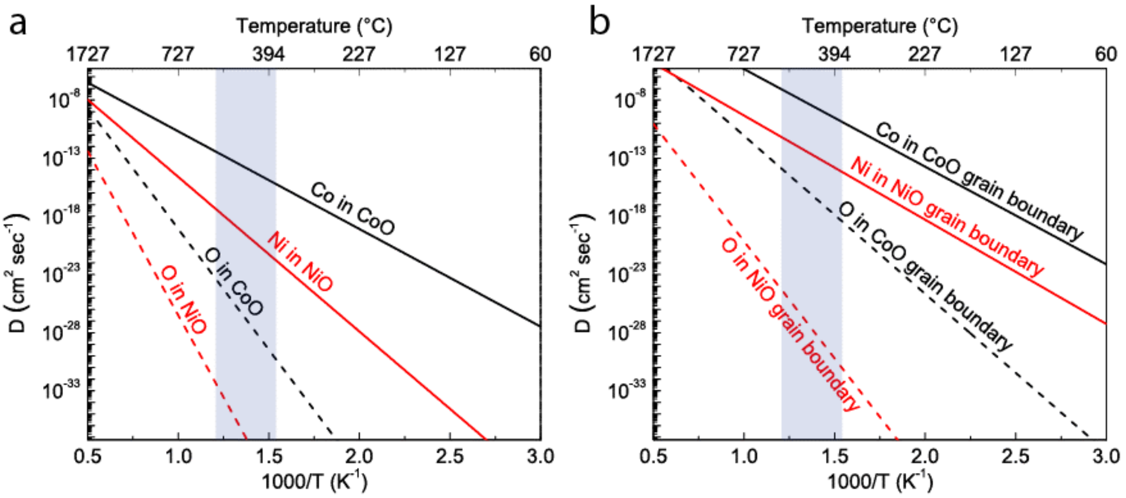

Supplementary Figure 6 | Comparison of temperature-dependence diffusion coefficient of O in CoO and O in NiO with that of Co in CoO and Ni in NiO. (**a**) Bulk diffusion; (**b**) grain boundary diffusion. The areas shadowed in blue correspond to the reaction temperature in our experiments.

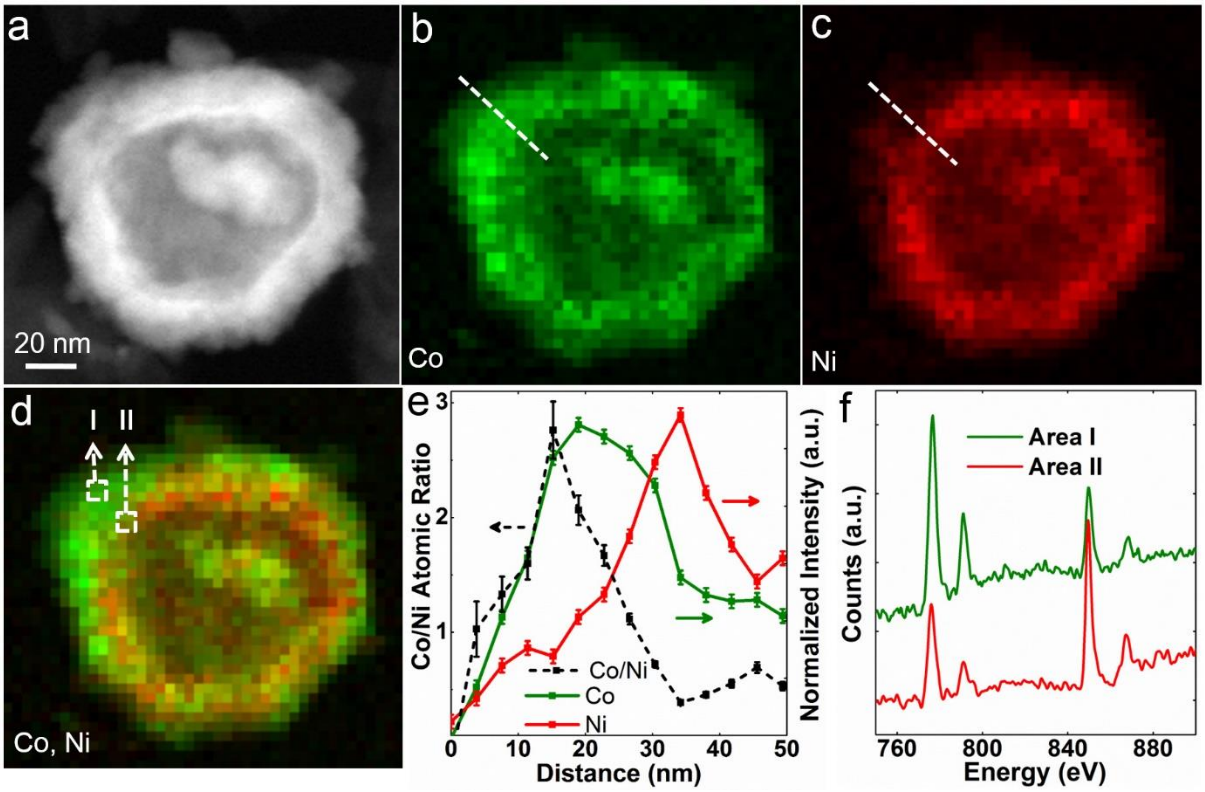

Supplementary Figure 7 | Detailed analysis of Ni and Co distribution in Ni_2Co oxidized at 400 °C in air. (**a**) STEM image; (**b**) Co color map; (**c**) Ni color map; (**d**) Co and Ni mixed-color map; (**e**) Co and Ni intensity distribution and Co/Ni atomic ratio distribution along the dotted lines in (**b**) and (**c**); (**f**) EELS spectra from the areas I and II in (**d**). The qualitative results in (**e**) were obtained according to the following procedures. First, we integrate over the full particle to obtain a low-noise spectrum. Second, we calculated the atomic ratio between Co and Ni using the EELS Quantification plugin in the Digital Micrograph software. To determine the ratio accurately, only edge signals 45 eV above the edge onset were used to obtain the cross section of the $L_{2,3}$ white lines correctly. We denote the ratio as $R_{continuum}$. Third, we calculate the intensity ratio of the Co L_3 white line and the Ni L_3 white line and we denote this ratio as R_{L3}. Forth, we can obtain a correction factor $C = R_{continuum}/R_{L3}$. Fifth, the atomic ratio between Co and Ni of each individual spectrum in the map can be determined by calculating the intensity ratio between the Co L_3 white line and Ni L_3 white line and multiply that by the correction factor C. The error in the extracted EELS signal is primarily determined by the uncertainly of the power-law background subtraction. In most EELS maps, Poisson noise is a small perturbation compared with the background subtraction error. The best way to estimate the error bar for edge intensity maps is to find an area where the desired element is not present and calculate the standard deviation of the map intensities. We have done this for Co and Ni respectively and the 95% confidence intervals (two times of the standard deviation) have been added to the plot. For the Co/Ni ratio plot, we have propagated the error using the equation $\Delta\left(\frac{x}{y}\right) = \sqrt{\left(\frac{\Delta x}{y}\right)^2 + \left(\frac{\Delta y * x}{y^2}\right)^2}$.

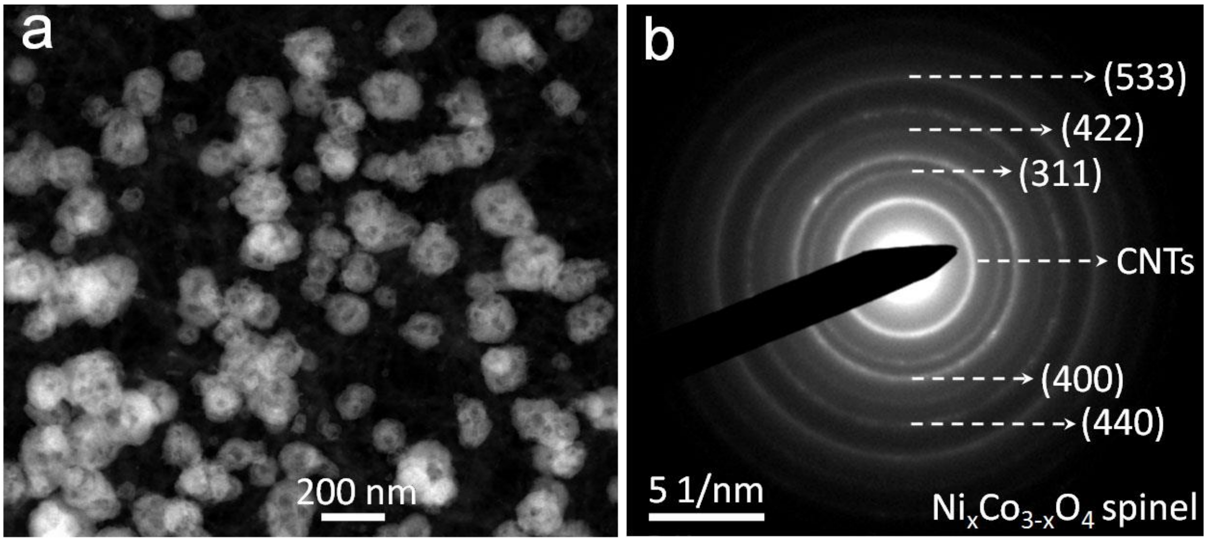

Supplementary Figure 8 | Characterization of morphology and crystal structure of the Ni_2Co sample after complete oxidation in air. (**a**) STEM image; (**b**) SAED pattern. The SAED pattern suggests the product of the oxidation process is the spinel structure.

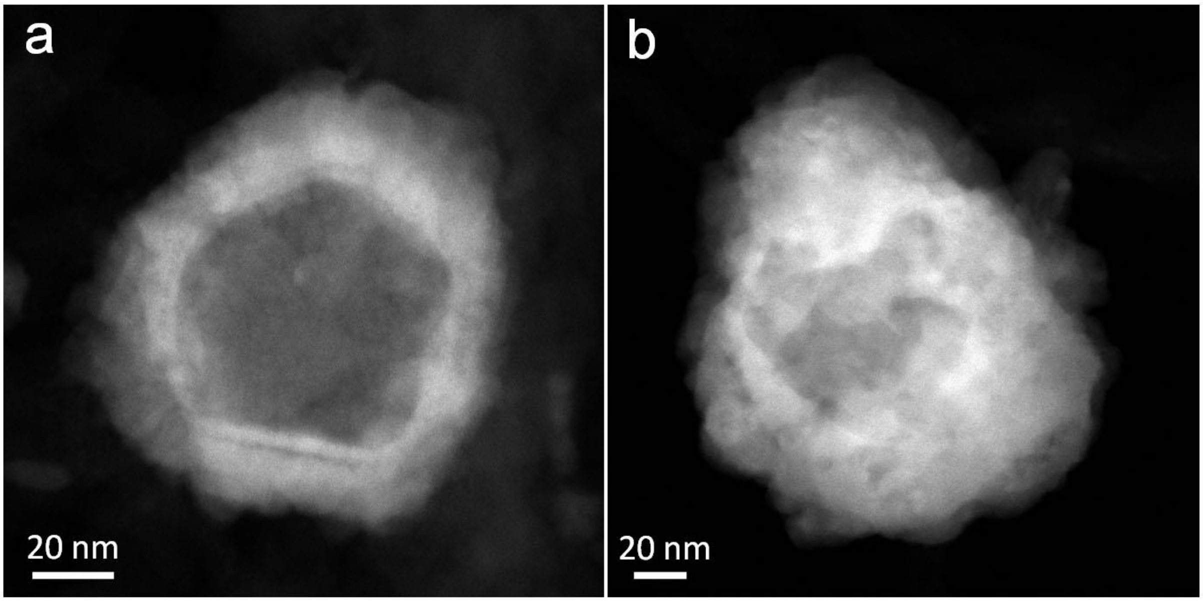

Supplementary Figure 9 | STEM images of two types of particles in the sample after complete oxidation in air. (**a**) Type I; (**b**) Type II.
